# The Influence of Various Occupations upon the Eye

**Published:** 1875-08

**Authors:** Lucian Howe


BUFFALO

Medical and Surgical Journal.

VOL XV. AUGUST, 1875. No. 1.

### Original Communications.

:o:

ART. I.—*The Influence of various Occupations upon the Eye.* By LUCIEN HOWE, M. D. Read before the Buffalo Medical Association, July 6th, 1875.

In the relation in which it is proposed to consider these different employments, they may be divided into four classes, according as they subject the eye to danger arising,

1. From injuries from foreign bodies.
2. From irritation of the conjunctiva.
3. From overtaxing the power of accommodation, and
4. From weakening the nervous sensibility.

Concerning each of these divisions, mention will be made of the persons included in them, the form of disease resulting, and the general indications for its treatment. Let us begin then with those individuals who are liable to injuries from foreign bodies. Of course, these accidents can happen to any one, but of those particularly exposed, there should be specified before all others that class of artizans who have to do with the manufacture or use of steel instruments.

In the process of forging them there is a shower of sparks driven from beneath the hammer, which carry destruction to any part of the eye they happen to touch. To every blacksmith these would be a source of constant danger, but for the fact that as he strikes the heated mass, they fly off at the side instead of in the direction of his face. When the instruments are to be burnished or sharpened on an emery wheel or grindstone, there is another risk to be run from the bits of stone and metal which then come off in the direction of the workmans eyes. Again, in the use of the chisel in cutting iron or copper, and by marble workers, and stone cutters in general, there is danger, not from the fragments driven off in front, but from those which rebound from beneath the point and which are often called "back chips." The number of accidents thus occurring is realized only by those whose attention, as specialists, is directed to the subject. An eminent German observer, has collected statistics which show that almost one-half of the workers in metal, suffer from such injuries.* In order to disprove or verify this statement, I have collected some figures bearing on the subject, and in general find the conclusion quite correct. Without going into detail it is necessary only to say that four of the largest establishments in this city were visited where various kinds of "edged tools" were manufactured, and where, of course, emery wheels and grindstones were used extensively. Among the blacksmiths, injuries to the eye were not found particularly frequent, but when they did occur were of a serious nature. Of the men engaged in sharpening or burnishing the instruments however, all, had suffered to a greater or less extent from the flying sparks of metal or bits of stone. In most instances these have been readily removed by some fellow workman, while a few were given over to the surgeon. For example, of 23 men so employed, during one year, each one was, on the average obliged to loose a day's work in every two months, two had been disabled for a longer time than a week, and one had lost an eye entirely. Next to this class of artizans, the persons most liable to similar accidents, are sportsmen. They arise sometimes from the premature explosion of gunpowder, but usually they come from the bursting of the percussion cap, fragments of which almost invariably enter the right eye, that one being nearer the hammer and open, while the other is closed in the act of aiming. In military practice however, such accidents

* Comp. Stellwag on the Eye, p. 16.

are rare, for the reason that the caps are better in quality and make than are those sold in the shops for ordinary or inferior guns.* Besides the individuals thus mentioned, of course any one is liable to accidents where bits of glass, wood, coal or other substance strike the eye with such force as to rupture its coverings and find lodgment within. These, and all other materials may be divided clinically into the two classes, of those which are, and which are not readily oxidizable. This distinction is a practical one for the reason that those of the former kind, such as fragments of iron, copper, wood and the like, must be removed from the eye immediately, and at all hazards, while it often seems so patient under irritation produced by minute particles of glass, charcoal, gunpowder, or even lead, that active measures are ill-advised. In all cases of course the wound must be closely watched, but in those where it heals externally, and symptoms of irritation subside, it is safe to conclude that either the foreign body is not in a position to do any immediate harm, or is not lodged in the eye at all. In instances where it is thus present, it becomes encapsuled and may remain for years without doing further injury.† For instance, the writer has notes of a case observed at the General Hospital in Vienna, where a small shot had penetrated to the center of the vitreous and could be seen there, covered with an apparent exudation, and having various attachments. In regard to the prophylactic treatment of the individuals mentioned as particularly exposed to the accidents under consideration, several methods have been proposed. The only practical one is the use of spectacles made of thin plates of mica.‡ This has the advantage over glass of being lighter, cheaper, and not so easily broken. Possibly, if they were better known to the workingmen of this country they would be more generally worn, but so far, even the safety afforded by ordinary glasses, is for the most part, disregarded.

To the second class of occupations which we shall consider, belong those persons who are habitually in an atmosphere which acts mechanically or chemically as an arritant to the conjunctiva. This excitation is produced by particles of matter finely disintegrated;

* Lawson, Diseases and Injuries of the Eye, p. 320.
† Max Tetzer, Compendium der Augenheilkunde, s. 312.
‡ Dr. Cohn, Berliner Klinishe Wochenschrift, Feb. 24, 1868.

it may be, as a mixture of various substances called collectively "dust;" it may be of carbon under the form of smoke, or of water as steam. Of the first, the most familiar example is the street dust, which in some localities fills the eyes of those driving or walking, producing sensations anything but agreeable. This is fortunately a cause which does not act sufficiently long to result in anything serious. Quite otherwise is it however, in mills for grinding corn, or wheat, or where other substances are reduced to a state of powder by filing, rasping, etc., and where persons engaged in the business are obliged to remain for hours in an atmosphere hardly respirable. In the same category, come those who labor in an air often dense with smoke, as employees on railway trains, together with those who work in rooms filled with steam, as laundresses, dyers and bleachers. Washerwomen for instance have often been noticed to suffer from conjunctivitis and in forms occasionally so intense, as to lead to the suspicion that it was communicated by some contagious secretion on the soiled clothes. Thus Galezowski* devotes an article to the precautions which these people should exercise in that respect. My own opinion is however, that it is due simply to the continual exposure of the eyes to the vapor and heat in the various rooms and the sudden changing from one to the other. A visit to a number of city laundries in this shows the methods of ventilation wonderfully imperfect. To a similar set of causes can be ascribed the conjunctivitis which sometimes attacks those engaged in boiling soap. In this process there is also one stage where the minute particles of slacking lime act as a chemical irritant, but in general, it is steam and heat that does the injury. Upon making inquiries regarding the frequency of such troubles, I find a proportion of over one-fifth in which chronic conjunctivitis shows itself in the eyes of those constantly in the "boiling room." The degree varies, however, with the susceptibility of the individual and the degree of incompleteness shown in ventilation. In a like manner, are dyers and bleachers exposed, but to what extent and with what effect, I am not aware. The number of persons liable to suffer from an atmosphere chemically injurious is naturally less, still they are to be found in labora-

*Traite des Maladies des eux, p. 876.

tories, mines and sewers. When the gasses exist in these places in a diluted form, they act as irritants simply, but if concentrated may have the same caustic effect as the lime does in the eyes of whitewashers and plasterers.

In regard to the question of treatment, all such patients should, of course, make it their first care to avoid as much as practicable the cause of the disease. But whether they continue at their work or not, the usual astringent solutions of zinc, copper or tannin should be used. Together with this, it is an excellent plan to frequently wash out the string of mucous which collects in a roll between the lids and globe. It is well to vary the temperature of the water thus employed with the form of the irritating cause. In cases where this has been accompanied by heat, as in smoke or steam, the water applied should be cool, otherwise lukewarm.

The third class of employments comprises those, where there is a tendency for persons engaged in them to overtax the power of accommodation. The greater part are occupied in reading or writing and those who are not, have to do with small objects at a short range of vision. Here are to be included the school children, ministers, lawyers and physicians, copying clerks, book-keepers and the like, together with watchmakers, engravers, etc.

The effect of thus straining the accommodation manifests itself in the lens, retina, or both. In the former it has been thought to be an element tending to produce cataract. It is true that statistics are wanting on this point, but one of the best authorities in France* says he is convinced that "with those accustomed to reading, writing, engraving, etc., opacities in the crystalline almost invariably commence in the inferior and internal quadrant, while with farmers * * * the opacity begins either in the nucleus or in the periphery." Overtaxing the accommodation often manifests itself also in a feeling of fatigue in the eyes, a redness of the conjunctiva and increased flow of tears—symptoms, which, taken together, constitute asthenopia. But by far the most frequent result is the development of myopia (near sightedness). This fact is of such importance that it is worth while to dwell more particularly upon it both as to cause and prevention. In the majority of

*Galezowski—Traite des Maladies des Yeux, p. 411.

instances the myopia is brought on during school life as has been shown by the statistics of Dr. Cohn* from an examination of over ten thousand children. He then proved conclusively that it is due to badly arranged desks, poor light and other unfavorable conditions. When the disease occurs in late life it again follows the use of books; and in general, it may be set forth as a rule, that the degree of literary culture arrived at by any nation or class of individuals is in direct proportion to the number of myopic persons there found. This is an observation long since made by writers on biology† and ethnology, and upon which especial stress has been laid by Donders.‡

Before indicating the prophylatic measures to be taken in guarding against this condition, a few words should be said concerning the anatomical changes accompanying it.

The causes of the disease will suggest its treatment. In myopia—especially that form called progressive myopia—it is well known that the eye is lengthened from before backward, a condition named posterior staphyloma—the rays of light passing through the lens are then focused in front of the retina instead of upon it. Now this lengthening of the globe is produced by pressure from within or without. Intra-ocular pressure occurs whenever an unusual amount of blood is present in the nutrient arteries whether sent there by the force of gravity or by mental excitement—a stooping posture, or the act of weeping, are instances which illustrates the temporary effect of over distension of the globe.

Again, the tension may be increased by muscular action, when the eye is adjusted for a point in the distance, the four recti muscles exert an equal traction, but if it converge toward its fellow, as in the act of accommodation, then the external rectus is, so to speak, bent around the globe and presses unduly upon it.

Let us now apply these two principles to the treatment of, or the precautions against myopia, and see what the practical deductions are; intra and extra-ocular pressure will be found to originate in unnatural postures and light,§ for the reason is evident, to sit in a

*Untersuchung der Augen von 10,060 Schul Kindern.

†Herbert Spencer's Principles of Biology, Vol. I, p. 249.

‡ Accommodation and Refraction of the Eye, p. 342.

§Donders—Refraction and Accommodation of the eye, p. 419.

high chair at a low table, and bend over one's book in a stooping position will give to any eye, not unusually strong, a feeling of weight and discomfort. There has been blood pumped down the arteries faster than it could flow up the veins and an over-distension results. Intra-ocular pressure is then avoided by simply sitting erect. The lesson regarding muscular action is quite as practical. When the light is dim we bring objects nearer in order to see clearly, the nearer the object the more the eye conveys, and the greater the convergance the greater the tension. In the next place therefore we should have sufficient light. But this does not mean a dazzling blaze shining full in one's face. The light should come from above or behind, and if artificial, should be without glare or flicker. A grateful protection against these latter qualities is furnished by the lamp shades now commonly used, sometimes, however, they have the advantage of being colored, and of the various tints, it has been found by experiment, that the least objectionable is blue, while the most irritating are yellow or orange.*

In addition to a good position for reading and the proper amount of light, there is a third precaution to be taken whenever a feeling of fatigue is experienced in the eyes. It is to give them a moments rest. The accommodative apparatus requires relaxation from work as much as any other set of muscles in the body.

If, in spite of all care, symptoms of myopia show themselves, then the patient should not be afraid of wearing glasses too soon. The popular prejudice in this respect is unfounded.† A pair that will enable one in such a condition to read ordinary type at twelve or fourteen inches distant is none to strong.

The last class of occupations which we shall consider is that in which there is a tendency to the lessening of the sensibility of the retina. This definition is of necessity as vague as are the causes of disease to which it refers. Such defects of vision are met with in sailors after long voyages, or in soldiers after exhausting campaigns, also in painters, looking-glass silverers and workmen in India rub-

*Stellwag on the eye, p. 19.

W. White Cooper—Practicll remarks on near and aged sight.

†Jefferies—The Eye in Health and Disease, p. 38.

ber factories.* In all cases the condition of the general health is much below par, and either presents the set of symptoms known as scorbutic, or else there are evidences of some particular systemic poisoning.

The dimness of vision thus resulting is called amblyopia, or when blindness is complete it is known as amaurosis. Two names these are which simply hide our ignorance regarding the anatomical changes giving rise to such symptoms. In fact, amaurotic effections may be defined as those in which the surgeon can not see anything abnormal, and neither can the patient see anything at all.

The treatment of such cases is constitutional and pre-eminently a matter of general principles. An important deduction concerning all these acquired imperfections is regarding their transmission from parent to offspring. That such instances do occur there can be no doubt, and of late many striking ones have been cited by authority beyond question in connection with the theory of evolution. Thus Bowman of London, has furnished Mr. Darwin with a number of such cases of inherited malformations and diseases of the eye.† At the same time the number of persons who are daily exposed to the dangers here enumerated, does not grow less. It seems then to be only a question of time before the tendency of civilized na'ions to the resulting affections, and especially to myopia, will be greatly increased, however, since the latter is, in most instances, contracted during school life, it becomes a matter worthy of serious consideration as to what measures can be adopted to obviate, as far as possible, the danger to which children in particular are subjected.

---

*Concerning the individuals so affected see

S ellwag—On the Eye, p. 773.

Wells—A Treatise on the Diseases of the Eye, p. 461

Hutchinson—On Lead Poisoning as a cause of Optic Neuritis.

Delpech—Memoire Sur Les Accidents que developpe chez les ouvriers en caoutchouc * * Bul de l'Acedmie de Med.

†For these and other examples see

Darwin—Animals and Plants under Domestication, Vol. 2, p. 17; also

Huxley's review of the Origin of Species.

